# Methods of Analysis in Randomized Noninferiority Trials: Methodological Survey Review Protocol

**DOI:** 10.2196/76876

**Published:** 2026-03-13

**Authors:** Daniel Xie, Sameer Parpia, Tyler McKechnie, Phillip Staibano

**Affiliations:** 1 Department of Integrated Biomedical Engineering and Health Sciences, Faculty of Health Sciences, McMaster University, Hamilton, ON, Canada; 2 Department of of Health Research Methods, Evidence, and Impact, Faculty of Health Sciences, McMaster University, Hamilton, ON, Canada; 3 Department of Oncology, Faculty of Health Sciences, McMaster University, 711 Concession St G-Wing Room 125, Hamilton, ON, L8V 1C3, Canada, 1 (905) 527-2299 ext 42685; 4 Department of Surgery, Faculty of Health Sciences, McMaster University, Hamilton, ON, Canada; 5 Department of Surgery, Division of Otolaryngology–Head and Neck Surgery, Faculty of Health Sciences, McMaster University, Hamilton, ON, Canada

**Keywords:** noninferiority, analysis population, analysis, randomized controlled trial, intention-to-treat, per-protocol

## Abstract

**Background:**

Noninferiority (NI) trial designs that investigate whether an experimental intervention is no worse than the standard of care have been used increasingly in recent years. The robustness of the conclusions depends in part on the analysis population set used. In NI settings, the intention-to-treat (ITT) and per-protocol (PP) analysis sets are most common. The ITT analysis has been considered anticonservative compared with the PP analysis.

**Objective:**

This study aimed to conduct a methodological review assessing the analysis population sets used in contemporary NI trials.

**Methods:**

A comprehensive electronic search strategy will be conducted to identify studies indexed in MEDLINE, Embase, Emcare, and Cochrane CENTRAL. Studies will be included if they are NI trials published in 2024. The primary outcome is the analysis population used for the primary analysis of the trial (ITT, PP, or as-treated). Secondary outcomes include the NI margin, effect estimates, point estimates, and corresponding CIs. Analyses will be performed using descriptive statistics.

**Results:**

The comprehensive search initially identified 1209 studies, of which 403 trials were eligible for data extraction. Data extraction began in January 2025 and is expected to be completed in January 2026.

**Conclusions:**

This methodological survey of NI trials will describe the analysis population used in the primary analysis and assess factors that may be associated with each analysis method.

## Introduction

In biomedical research, randomized controlled trials (RCTs) are generally considered the gold standard for comparing a new treatment with the standard of care (SOC) [1]. Superiority RCTs determine whether a new treatment is more efficacious than the SOC [1]. However, a new treatment may provide similar therapeutic effects while providing additional benefits, such as improved convenience, lower cost, or fewer adverse effects [2]. In such cases, noninferiority (NI) trial designs are used to demonstrate that a new treatment is no worse than the SOC [2] by a prespecified NI margin, which represents the maximum acceptable loss of efficacy of the new treatment relative to the SOC in exchange for the benefits of the new treatment [3].

While NI trials have their benefits, they also present unique challenges, as they require specific trial design, analysis, and interpretation. Researchers continue to debate the optimal application of intention-to-treat (ITT), per-protocol (PP), or as-treated (AT) populations when analyzing NI trials [4]. ITT analysis is preferred in superiority trials because of its conservative nature. Inclusion of patients who are nonadherent to the study protocol tends to attenuate treatment effects toward the null. However, in NI trials, this same characteristic may increase the risk of researchers falsely concluding there is no difference between the treatment and control groups (type I error in the NI setting), because the objective is to demonstrate that the investigational treatment is not worse than the SOC [5].

PP analysis is designed to assess the effect of an intervention among patients who adhere to the study protocol. However, excluding participants after randomization can introduce either positive or negative bias, which may lead to incorrect conclusions [6,7]. Despite this limitation, PP analysis is often used in NI trials, as it is generally considered to be less affected by protocol deviations [8].

Recent reviews of NI trials [9-12] do not adequately capture contemporary trials published after the CONSORT-Outcomes 2022 extension of the original CONSORT 2012 statement, which recommends that authors more clearly define the analysis population used for each outcome and explain how protocol nonadherence is accounted for in the trial protocol [13]. In practice, there is often a lag between the publication of new research guidelines and their consistent application; implementation can take over a year as researchers become aware of and adopt new practices. By analyzing trials published in 2024, we aim to capture trials that have implemented the 2022 CONSORT-Outcomes extension.

Current recommendations suggest using both the ITT and PP analyses, under the assumption that the ITT analysis is anticonservative in the NI setting [13]. The largest previous review focused on trials published in five leading medical journals (*The New England Journal of Medicine*, *The Lancet*, *Journal of the American Medical Association*, *Annals of Internal Medicine*, and *The BMJ*) [7], thereby limiting the generalizability of its findings. Lastly, it remains unclear whether the number of participants excluded from the PP population affects the choice of the primary analysis set.

Therefore, we aim to conduct a methodological review investigating the primary analysis population used in NI trials published in 2024 and whether this choice is associated with the proportion of patients with protocol deviations. Understanding current practices in the contemporary literature will help guide the future design of NI trials.

## Methods

### Study Design

This study is a methodological survey of contemporary NI trials published across all medical and surgical disciplines. The methodology for this review is reported in accordance with PRISMA-P (Preferred Reporting Items for Systematic Review and Meta-Analysis Protocols) (Checklist 1). The study protocol was registered with the International Prospective Register for Systematic Reviews (PROSPERO; CRD420251021089).

### Primary Research Question

What types of analysis population sets (eg, ITT/mITT (modified ITT), PP, ITT and PP, AT, other) are used for the primary analysis in NI trials published in peer-reviewed medical journals between January 1, 2024, and December 31, 2024?

### Secondary Research Objective

This study aimed to evaluate the relationship between the primary analysis population set for NI trials and protocol adherence.

### Eligibility Criteria

The eligibility criteria are outlined in Textbox 1.

Textbox 1. **Inclusion and exclusion criteria.**

**Inclusion criteria**

- Peer-reviewed noninferiority randomized controlled trials defined as a study self-described as a noninferiority trial in their title, abstract, introduction, or methods of publication.
- Published from January 1, 2024, to December 31, 2024, in any medical or surgical discipline.
- Published in English.

**Exclusion criteria**

- Studies described as pilot or feasibility randomized trials.
- Secondary analyses of randomized trial data or any other type of study not reporting primary data.
- Conference presentations or abstracts.
- Cluster noninferiority trials.

Since this work is conducted by researchers in Canada and is unfunded, we do not have the resources to invest in software to accurately translate articles published in multiple languages. Therefore, feasibility and resource constraints prevent us from expanding our search to include publications in other languages. We focused on individual randomized trials because cluster randomized trials may have low adherence at both the unit and individual levels, which adds complexity to the definition of ITT and PP analyses in cluster randomized trials.

### Information Sources

The following databases were searched from January 1, 2024, to December 31, 2024: MEDLINE, Embase, Emcare, and Cochrane CENTRAL. References of studies meeting the inclusion criteria were manually searched to ensure that all relevant articles were included.

### Search Strategy

The search strategy was designed with input from the study investigators and a medical research librarian. The Medical Subject Heading term “non-inferiority” was used for each database. Search terms related to NI trials such as “noninferiority randomized trial,” “noninferiority design,” and “noninferiority clinical trial” were also used. A full list of search strategies is available in Multimedia Appendix 1.

### Study Selection

Two reviewers will independently evaluate the searched titles and abstracts using Covidence systematic review software (Veritas Health Innovation Ltd). Each reviewer will be adequately trained and familiarized with the inclusion and exclusion criteria to ensure accurate and consistent screening. Discrepancies at the title and abstract and full-text screening stages will be resolved by consensus. If disagreement persists, an additional reviewer will be consulted. Interrater reliability will be measured with the Cohen κ statistic. This statistic is more informative than the proportionate agreement statistic because it adjusts for agreement occurring by chance [14].

### Outcome Definitions

The primary outcome for this study will be the analysis population set used in the primary analysis of the NI trials.

### Data Management

Two reviewers will independently conduct data extraction into a data collection form designed a priori. Discrepancies will be reviewed in detail by a third reviewer, who will resolve any conflicts. A piloted Microsoft Excel file of the extraction form can be found in Multimedia Appendix 2. The extracted data will include the following:

- Trial characteristics, including author, year of publication, journal of publication, specialty, study period, intervention details, and control details
- Number of patients randomized
- Protocol adherence, including number of patients who crossed over, number of patients who were nonadherent to the assigned intervention, and number of patients lost to follow-up
- Analysis population used in the primary analysis
- Outcome data in each analysis population, including type of primary outcome (continuous, binary, or time of event), direction of outcome (positive or negative), reported point estimate and 95% CI, reported effect estimate and 95% CI, number of primary outcome events (if binary), mean (SD) of the primary outcome (if continuous), and method of statistical analysis for the primary outcome
- Trial conclusion, including inferior, NI, superior, or inconclusive

### Statistical Analysis

The analysis population method used in the primary analysis in the trials will be summarized descriptively (ie, the proportion of studies using each method) and corresponding 95% CIs will be estimated using the Wilson score method [15]. Logistic regression will be used to investigate the relationship between the primary analysis population and protocol adherence by regressing the outcome against the proportion of patients in the ITT population who are excluded in the PP analysis, modeled using restricted cubic splines [16]. All statistical analyses will be performed using Stata (version 18; StataCorp LLC).

### Risk of Bias Assessment

Risk of bias assessment will not be performed for this methodological review. Risk of bias assessment is not recommended for methodological reviews, because the goal is to understand the methods applied in the studies being assessed [17].

### Dissemination

The findings of the survey will be submitted for publication in a peer-reviewed journal aimed at better informing clinical investigators on the current state of NI trial design. The results will be presented at conferences pertaining to clinical trial methodology. Study findings will also be communicated to relevant stakeholders (eg, investigators, biostatisticians).

### Ethical Considerations

Ethics approval was not required because this is a methodological survey based entirely on previously published clinical trials.

## Results

A total of 2102 studies were captured in the original search, and 893 duplicates were removed. Thus, 1209 studies were available for title and abstract screening. As of December 2025, this review is in the data extraction phase, with 403 studies eligible for extraction. Data extraction started in January 2025, and we expect results to be published in January 2026. Populated data extraction sheets for each included trial will be attached as supplemental files to the final manuscript to promote open science practices.

## Discussion

We anticipate that most trials will use the ITT and PP analysis population sets to adhere to the updated CONSORT-Outcomes 2022 extension for NI trials. Based on our initial data extraction, we hypothesize that the ITT will be the most prevalent analysis set despite its tendency to attenuate the treatment effect toward the null, thereby increasing the risk of a type I error. For this reason, we further hypothesize that trial nonadherence will be correlated with the use of the PP analysis set, as the PP set may better reveal true inferiorities that could be masked in an ITT analysis.

Uncertainty regarding which analysis population set should be used for NI trials is ubiquitous in the contemporary literature. Despite the methodological concerns associated with using the ITT population in the NI setting, several recent reviews have found that ITT remains the most prevalent analysis population set. A 2021 review of antibiotic NI trials published before 2019 found that 72.2% (164/227) of studies used both ITT and PP analyses, 18.2% (41/227) of studies used ITT analysis, and 9.7% (22/227) used PP analysis [9], suggesting that when a single analysis population is designated as primary, ITT is the most common. A 2020 review of oncological NI trials published from 2012 to 2018 found that 50% (55/110) of trials reported using only one analysis population, and 87.3% (48/55) of those used the ITT population [10], further reinforcing that ITT is most commonly used when a single primary analysis population is specified. Most recently, a 2024 review evaluating the methodological quality of NI trials published from 2014 to 2019 also found that most trials used both ITT and PP analyses [11]. It is important to note that all of these reviews examined trials published before 2022. A limitation of these reviews is that they do not report how often each analysis set was designated as the primary, an aspect our study will offer.

In 2022, the CONSORT-Outcomes 2022 extension of the CONSORT 2012 statement was published, recommending that authors further explain the definition of the analysis population used for each outcome and how it relates to nonadherence in the trial protocol [13]. A 2022 paper also suggested that the primary analysis in the NI setting should be the PP or AT populations, given the weaknesses of the ITT approach in NI trials [18]. NI trials published since then may demonstrate more robust reporting of the rationale for the analysis population sets applied, and there may be a shift away from the predominant use of the ITT approach observed in previous reviews.

In addition to assessing the prevalence of analysis population sets in contemporary NI trials, examining whether protocol adherence is associated with certain analysis populations will help researchers understand whether the decision to use the ITT or PP population is influenced by the proportion of patients who do not adhere to the protocol. No previous review has assessed whether an association exists between the primary analysis population set and protocol adherence among trial participants. This information will enable trialists to make more informed decisions regarding analysis population selection in NI trials, contributing to more rigorous and valid synthesis of health evidence. Once this review is completed, the findings will be used to inform a second study comparing the results of NI trials by their designated analysis population set to evaluate how outcomes differ.

A limitation of our study is sampling bias, as we are analyzing only NI trials published in 2024. We focused our review on trials published in 2024 to capture contemporary literature that may have implemented the 2022 CONSORT-Outcomes extension for NI trials. As a result, we will not be able to report longitudinal trends. A second limitation is that restricting our search to NI trials published in English reduces generalizability. Non-English trials may differ systematically in the research methods applied. Moreover, the most impactful papers are typically published in English-language journals with high impact factors [19]. This may bias the dataset because trials published in high-impact journals are more likely to be methodologically robust and to declare NI. For this reason, we designed a broad search strategy to ensure that we captured all NI trials published in peer-reviewed journals.

Despite these limitations, this study will provide valuable insights into the analytic approaches used in contemporary NI trials and the factors that may influence their selection.

## Supplementary material

10.2196/76876 Multimedia Appendix 1 Search strategy.

10.2196/76876 Multimedia Appendix 2 Extraction template.

10.2196/76876 Checklist 1 PRISMA-P checklist.

## Abbreviations

AT: as-treated
ITT: intention-to-treat
NI: noninferiority
PP: per-protocol
RCT: randomized controlled trial
SOC: standard of care
