# Supplementary material for: Methods of Analysis in Randomized Noninferiority Trials: Methodological Survey Review Protocol
Source: JMIR Res Protoc. 2026 Mar 13;15:e76876. doi: 10.2196/76876 (PMC12987403; doi:10.2196/76876)
Supplement: Multimedia Appendix 1 [file resprot-v15-e76876-s001.docx]

1. Equivalence trial/
2. (non-inferiority trial* or noninferiority trial* or non inferiority trial*).mp.
3. (non-inferior trial* or noninferior trial* or non inferior trial*).mp.
4. (non inferiority clinical trial* or noninferiority clinical trial* or non-inferiority clinical trial* or non inferiority design* or noninferiority design* or non-inferiority design*).mp.
5. (non inferior clinical trial* or noninferior clinical trial* or non-inferior clinical trial* or non inferior design* or noninferior design* or non-inferior design*).mp.
6. (randomised controlled non-inferiority trial* or randomized controlled non-inferiority trial or randomised controlled noninferiority trial* or randomized controlled noninferiority trial or randomised controlled non inferiority trial* or randomized controlled non inferiority trial).mp. [mp=title, book title, abstract, original title, name of substance word, subject heading word, floating sub-heading word, keyword heading word, organism supplementary concept word, protocol supplementary concept word, rare disease supplementary concept word, unique identifier, synonyms, population supplementary concept word, anatomy supplementary concept word]
7. or/1-6
8. Limit 7 to yr=“2024”

**Supplemental Table 2.** Complete search strategy (Medline database example)

1. Non-inferiority trial/
2. (non inferiority trial* or non-inferiority trial* or noninferiority trial*).mp.
3. (non inferior trial* or non-inferior trial* or noninferior trial*).mp.
4. (non inferiority clinical trial* or noninferiority clinical trial* or non-inferiority clinical trial or non inferiority design* or noninferiority design* or noninferiority design* or non-inferiority design*).mp.
5. (non inferior clinical trial* or noninferior clinical trial* or non-inferior clinical trial or non inferior design* or noninferior design* or noninferior design* or non-inferior design*).mp.
6. (randomised controlled non-inferiority trial* or randomized controlled non-inferiority trial or randomised controlled noninferiority trial* or randomized controlled noninferiority trial or randomised controlled non inferiority trial* or randomized controlled non inferiority trial).mp. [mp=title, book title, abstract, original title, name of substance word, subject heading word, floating sub-heading word, keyword heading word, organism supplementary concept word, protocol supplementary concept word, rare disease supplementary concept word, unique identifier, synonyms, population supplementary concept word, anatomy supplementary concept word]
7. Or/1-6
8. Limit 4 to yr=“2024”
9. limit 8 to "remove medline records"

**Supplemental Table 3.** Complete search strategy (Embase and Emcare database example)

1. Equivalence trial/
2. non-inferiority trial* or noninferiority trial* or non inferiority trial*).mp.
3. (non-inferior trial* or noninferior trial* or non inferior trial*).mp.
4. (non inferiority clinical trial* or noninferiority clinical trial* or non-inferiority clinical trial* or non inferiority design* or noninferiority design* or non-inferiority design*).mp.
5. (non inferior clinical trial* or noninferior clinical trial* or non-inferior clinical trial* or non inferior design* or noninferior design* or non-inferior design*).mp.
6. (randomised controlled non-inferiority trial* or randomized controlled non-inferiority trial or randomised controlled noninferiority trial* or randomized controlled noninferiority trial or randomised controlled non inferiority trial* or randomized controlled non inferiority trial).mp. [mp=title, abstract, heading word, drug trade name, original title, device manufacturer, drug manufacturer, device trade name, keyword heading word, floating subheading word, candidate term word]
7. or/1-6
8. non-inferiority trial/
9. (non-inferiority trial* or noninferiority trial* or non inferiority trial*).mp.
10. (non-inferior trial* or noninferior trial* or non inferior trial*).mp.
11. (non inferiority clinical trial* or noninferiority clinical trial* or non-inferiority clinical trial* or non inferiority design* or noninferiority design* or non-inferiority design*).mp.
12. (non inferior clinical trial* or noninferior clinical trial* or non-inferior clinical trial* or non inferior design* or noninferior design* or non-inferior design*).mp.
13. (randomised controlled non-inferiority trial* or randomized controlled non-inferiority trial or randomised controlled noninferiority trial* or randomized controlled noninferiority trial or randomised controlled non inferiority trial* or randomized controlled non inferiority trial).mp. [mp=title, abstract, heading word, drug trade name, original title, device manufacturer, drug manufacturer, device trade name, keyword heading word, floating subheading word, candidate term word]
14. or/8-13
15. 7 or 14
16. limit 15 to yr="2024"

**Supplemental Table 4.** Complete search strategy (CENTRAL database example)
